# Is obesity a predisposing factor for free flap failure and complications? Comparison between breast and nonbreast reconstruction: Systematic review and meta-analysis: Erratum

**DOI:** 10.1097/MD.0000000000006213

**Published:** 2017-02-17

In the article “Is obesity a predisposing factor for free flap failure and complications? Comparison between breast and nonbreast reconstruction: Systematic review and meta-analysis”,^[1]^ which appeared in Volume 95, Issue 26 of *Medicine*, the editor's name should have been reported as Goran Augustin.
